# Spatio-temporal characteristics of meteorological drought in Khyber Pakhtunkhwa, Pakistan

**DOI:** 10.1371/journal.pone.0249718

**Published:** 2021-04-15

**Authors:** Ghani Rahman, Atta-ur Rahman, Sami Ullah, Muhammad Dawood, Muhammad Farhan Ul Moazzam, Byung Gul Lee

**Affiliations:** 1 Department of Geography, University of Gujrat, Gujrat, Pakistan; 2 Department of Geography, University of Peshawar, Peshawar, Pakistan; 3 Department of Civil Engineering, College of Ocean Sciences, Jeju National University, Jeju, South Korea; Universiti Teknologi Malaysia, MALAYSIA

## Abstract

This study analysed spatio-temporal fluctuations in rainfall to assess drought and wet spells in Khyber Pakhtunkhwa, Pakistan. Temporal changes in rainfall were assessed using a linear regression method, while aridity conditions at each meteorological station were measured using the United Nations Environment Programme climate aridity index. In this study, drought and wet spell patterns were identified using the Standardized Precipitation Evapotranspiration Index (SPEI). The Spearman’s Rho (SR) test was applied to find trends in the temporal 1-month and 12-month SPEI data. Balakot, Dir, Kakul, Kalam, Malam Jabba, Parachinar, Patan and Saidu were humid whereas Cherat and Timergara were sub-humid meteorological stations while Bannu, Chitral, Drosh and Peshawar were semi-arid and D.I. Khan was found to be the only arid meteorological station in the study area. The regression results revealed that the amount of rainfall is decreasing at Balakot, Kakul and Dir, while in the southern part of the province the amount of rainfall is increasing, such as in Parachinar and Cherat. The SPEI results revealed distinct drought spells in 1971–1974, 1984–1989, 1998–2004 and recently in 2017–2018, in almost all met-stations results. The SR results indicated a significant wet trend at met-station Parachinar, located in the west, while a significant drying trend has been noted at Balakot in the north-eastern part of the study area. Detailed knowledge about rainfall variability can provide a foundation for the planning and use of water resources.

## Introduction

Drought is an insidious natural hazard with detrimental effects on the natural environment and human social systems [1]. The number of droughts, their extent, and severity have been steadily rising due to an increase in extreme climatic events and climatic instability [2]. Droughts are climatic anomaly directly related to prolonged and abnormal deficiency in rainfall [3, 4], resulting in water shortage for water-dependent activities [5, 6]. Measuring droughts commence, their extent, duration, and cessation is difficult because of their geographic and temporal variability [7]. Drought can distress large geographical areas, depending on the temporal and spatial extent of the precipitation deficit, as well as the drought’s severity [8]. Drought mostly results in non-structural damage over widespread regions [8, 9] thus does not receive as much attention as a flood and earthquake. Nonetheless, drought comparatively affects more people and territory than other natural hazards [6, 10, 11].

Scientists and policymakers are often hesitant to announce a drought event has begun, and its extent, because there is little agreement on a universally accepted definition, due to its diverse geographical nature [12]. Drought characteristics and impacts can vary significantly from region to region, and consequently droughts have been variously classified based on their meteorological, hydrological, agricultural and socio-economic features [13]. All droughts originate with a decline in rainfall. Other drought intensifying factors may also exist, such as winds, high temperature, and low humidity [14]. An unusual reduction in the amount of rainfall, or long periods of dryness, are considered meteorological drought, and its effects can cause agricultural drought [15]. Meteorological drought also precedes hydrological drought, which is characterized by a reduction in streamflow, water scarcity and insufficient available water for irrigation, an increasing rate of evapotranspiration, lowering groundwater table and reduced soil moisture level [6, 15].

The severity of drought in an area depends on the level of soil moisture deficiency and the extent of the event [6]. Long duration droughts deplete soil moisture and exhaust surface and subsurface water [15, 16], leading to famine as well as huge socio-economic and environmental damages. Drought frequency and severity may be further aggravated by changes in global temperature and rainfall patterns [16, 17]. To assess drought and quantify their severity and spatial extent, several indices have been developed [18]. The most commonly used indices are the Standardized Precipitation Index (SPI), Standardized Precipitation Evapotranspiration Index (SPEI), Palmer Drought Severity Index (PDSI), Reconnaissance Drought Index (RDI), Surface Water Supply Index (SWSI) and Effective Drought Index (EDI) etc. These indices are effectively used to assess and monitor drought in different parts of the world [7, 19].

South Asia is a recurrently drought-affected region of the world. In the last fifty years India, Pakistan, Afghanistan, and Sri Lanka have experienced droughts with a frequency of one every three years [20]. In Pakistan, except for the northern mountains, the climate is semiarid to hyper-arid and thus most parts of the country are highly prone to the threat of drought [21]. This situation is a consequence of the long latitudinal extent of the country. Most of the rain received in the months from December to March are from western disturbances, and in July to September from the summer monsoon, while the remaining five months receive an insignificant amount of rain [21]. The economy of the country depends mostly on agricultural products and most of these agricultural activities are dependent on rainfall because there is a limited amount of irrigation water available. Variation in the amount of rainfall can lead to disastrous impacts. In some years, the country faces disastrous floods due the high amounts of rainfall received in short periods of time, while deficiencies in the amount of rainfall lead to drought in the arid and semi-arid areas, which in turn affect the agriculture sector.

The purpose of this study is to assess the spatio-temporal characteristics of drought in Khyber Pakhtunkhwa (KP), Pakistan. In Khyber Pakhtunkhwa, rainfall varies widely in terms of occurrence as well as quantity and distribution [22]. Hazara, Malakand, and most of the Peshawar division receive their maximum rainfall from the monsoon in summer, while other parts of the province receive very little rain during this season [22]. Arid and semiarid conditions prevail in the southern and extreme northern parts of the study area, while the north-western and north-eastern are humid [22]. During the past forty-eight years, in KP province the dry spells have consistently increased, affecting the water potentially available for crops. The analysis of rainfall and evapotranspiration variability is of key significance in the area, where extreme dry conditions lead to drought.

## The study area

**Fig 1:**
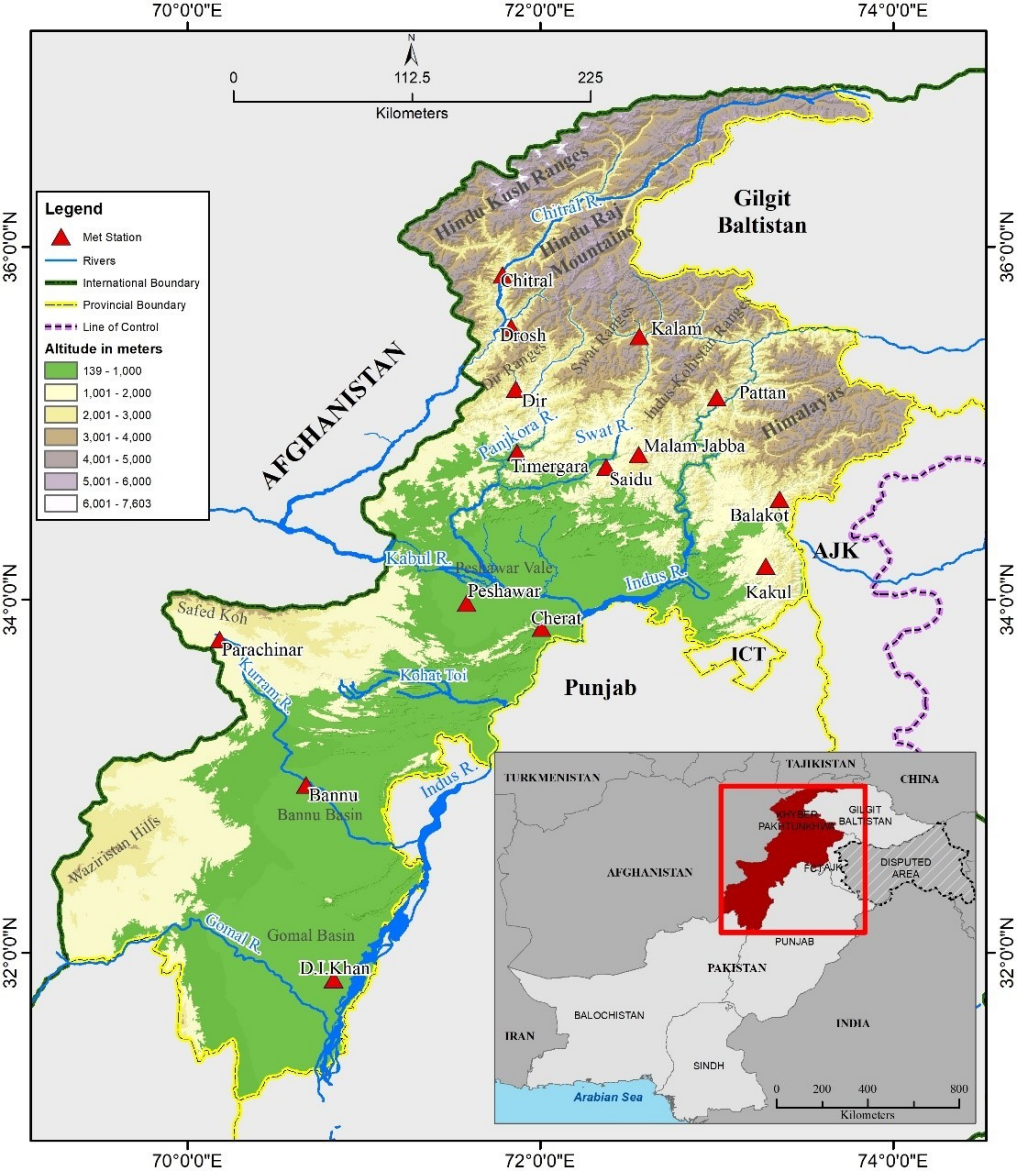
Khyber Pakhtunkhwa and location of meteorological stations in the study area.

The study area, KP is a province of Pakistan situated in the north-west of the country (Fig 1). Geographically, its latitudinal extent is 31° 24՜ to 36° 92՜ North and longitudinal extent is 70° 07՜ to 74° 14՜ East. The topography of the province varies from low-lying plains in the south to highest peaks of more than 7,000m in the north and northeast. Because of this variation in topography, the climate is also highly diversified. In the south winters remains mild, while extreme cold winters and mild summers prevail in the northern parts of the province [23]. The major rivers flowing through the province are the Indus, Kabul, Swat, Chitral, Kurram, Tochi and Gomal. These rivers provide water for irrigation. The high mountain chains of the Hindu Kush and Himalayas are situated in the north and northeast of the study area. The two major sources of precipitation in the region are monsoons in the summer and western depressions in winter as well as in spring.

The high mountains mostly receive precipitation in the form of snow in winter, which is a major source of water for the perennial river systems. Sufficient rains are received in the northern mountainous region while dry conditions mostly prevail in the plains areas of the province, due to less rainfall. A gradual decrease in rain and snowfall has been observed over the northern mountainous belt of the study area, which has affected water availability in rivers flowing in the province, and in turn has badly affected the agricultural sector (Khan et al., 2003). Agriculture is the mainstay of the economy in the country, contributing 38% to the GDP, while 44% of the population are directly employed in agriculture [22–25].

## Material and methods

### Data collection and methodology

To achieve the objectives of the study, data on temperature, precipitation, and hours of sunshine was collected from the Pakistan Meteorological Department for the period of 1971–2018. Linear regression was applied to assess the magnitude of the annual trend in rainfall data from each meteorological station. Temperature and rainfall data were used to calculate potential evapotranspiration and for drought assessment. Drought severity was assessed using the standardized precipitation evapotranspiration index (SPEI).

### Standardized precipitation evapotranspiration index (SPEI)

The SPEI calculations are similar to those in SPI and have been widely used to assess drought occurrences [26]. However, the SPI only requires monthly rainfall, while the SPEI calculation needs a monthly water balance, which is based on the difference between rainfall and potential evapotranspiration (PET). The PET calculation involves several parameters including surface temperature, relative humidity, the intensity of solar radiation on the earth surface, water vapour in the air, latent and sensible heat fluxes [27]. These meteorological data are not available from most the world’s meteorological stations. Therefore, to calculate PET, various other methods such as the Penman-Monteith method [27], Hargreaves method [28], and Thornthwaite method [29] have been proposed to estimate it indirectly from a few available meteorological parameters. In this study the Thornthwaite method was applied to calculate PET, because of the non-availability of data. The Thornthwaite method requires mean monthly temperature and monthly mean sunshine hours as the key factors. PET is calculated using the following methodology:

$$\mathrm{PET}=16\times \left(\frac{N}{12}\right)\times \left(\frac{m}{30}\right)\times {\left(10\times \frac{T_{i}}{I}\right)}^{\alpha }$$ (1)

In this equation, *N* denotes mean monthly sunshine hours while *m* expresses the number of days in a month, *Ti* is the monthly average temperature in °C, *α* is the coefficient depending on I:

$$\alpha =6.75\times 10^{-7}\times I^{3}-7.71\times 10^{-5}\times I^{2}+1.79\times 10^{-2}\times I+0.49$$ (2)

In this equation, *I* expresses the thermal index derived from the sum of the 12-month index values using the following expression:

$$I=\overset{12}{\underset{i=1}{\sum }}{\left(\frac{T_{i}}{5}\right)}^{1.514}$$ (3)

After obtaining the PET value using Eq (1), the difference between the precipitation and PET (water balance) was derived based on the following expression:

$$D_{i}{=P}_{i}{-\mathrm{PET}}_{i}$$ (4)

The *D*_*i*_ is the water balance derived from the difference between precipitation and PET, which indicates a surplus or deficit of water for the analysed month. The *D*_*i*_ results are accumulated at different time scales, using the following equation.

$$D_{n}^{k}=\overset{k-1}{\underset{i=0}{\sum }}\left(P_{n-i}-PET_{n-i}\right),\;n\geq k,$$ (5)

In this equation, *k* expresses the time scale of the data and n is the calculation frequency. SPEI needs three parameters distribution, Pearson III, lognormal and extreme values, and this differentiates it from SPI, since SPI uses two parameters (Vicente-Serrano et al., 2010; Yang et al., 2016). The variable x has a lower value boundary of zero (0>*x*<∞) in the two parameters distribution, while in the three parameters distribution the x can take values in the range (*γ*>*x*<∞), where the *γ* is the parameter of origin of the distribution. The variable x can have negative values and the existence of a negative value is common in *D* series. To model the *Di* values, the three parameters, log-logistic probability density function was applied.

$$f(x)=\frac{\beta }{\alpha }{\left(\frac{x-\gamma }{\alpha }\right)}^{\beta -1}{\left[1+\frac{x-\gamma }{\alpha }\right]}^{-2}$$ (6)

In this equation, the *α* is the scale parameter, *β* is a shape parameter and *γ* is the origin parameter obtained using the *L*-moment procedure through the following equations:

$$\alpha =\frac{\left(w_{0}-2w_{1}\right)\beta }{\Gamma (1+1/\beta )\Gamma \left(1-\frac{1}{\beta }\right)}$$ (7)

$$\beta =\frac{2w_{1}-w_{0}}{6w_{1}-w_{0}-6w_{2}}$$ (8)

$$\gamma =w_{0}-\alpha \Gamma \left(1+\frac{1}{\beta }\right)\Gamma \left(1-\frac{1}{\beta }\right)$$ (9)

where Γ(*β*) is the gamma function of *β*. The following expression gives the probability distribution function of the log-logistic distribution for D series data:

$$F(x)={\left[1+{\left(\frac{\alpha }{x-\gamma }\right)}^{\beta }\right]}^{-1}$$ (10)

With *F*(*x*) the SPEI can easily be calculated as the standardized values of *F*(*x*) with the following equation:

$$\mathrm{SPEI}=W-\frac{c_{0}+c_{1}W+c_{2}W^{2}}{1+d_{1}W+d_{2}W^{2}+d_{3}W^{3}}$$ (11)

where $W=\sqrt{-2In(P)}\;for\;P\leq 0.5$ where *P* is the probability of exceeding a determined *D* value, *P* = 1−*F*(*x*); when *P*>0.5, *P* = 1−*P* and the constant values are *C*_0_ = 2.515517, *C*_1_ = 0.802853, *C*_2_ = 0.010328, *d*_1_ = 1.432788, *d*_2_ = 0.189269, and *d*_3_ = 0.001308.

The zero value is an average of SPEI. Positive values indicate above-normal precipitation in the studied area, while negative values denote a drought situation (Table 1). The SPEI scale is as follows:

**Table 1:** Drought and wet categories based on SPEI values.

Category	SPEI Value
**Extreme wet**	≥ 2
**Severe wet**	1.5 to 1.99
**Moderate wet**	1.0 to 1.49
**Mild wetness**	> 0 and < 1
**Normal**	0
**Mild dryness**	< 0 and >-1
**Moderate drought**	-1.0 to -1.49
**Severe drought**	-1.5 to -1.99
**Extreme drought**	≤ -2.0

### Spearman’s Rho trend test

Different statistical methodologies can be used to identify trends in the time series data, and each method has its pros and cons [30]. In this study, the Spearman’s Rho (SR) trend test is applied to find trends in the SPEI results. It is one of the commonly used trend test applied for times series analysis. It is a rank-based nonparametric method to determine the presence or absence of a trend in time series data. It is based on two hypotheses: the null hypothesis (H_0_) which states that all the temporal data are independent and identically distributed. The alternative hypothesis (H_1_) means there is either an increasing or decreasing trend in the data. The SR trend test statistic D and the standardized test statistic *Z*_*SR*_ are statistically derived as:

$$D=1-\frac{6\sum_{i=1}^{n}{\left(R_{i}-i\right)}^{2}}{n(n^{2}-1)}$$ (12)

$$Z_{SR}=D\sqrt{\frac{n-2}{1-D^{2}}}$$ (13)

In Eq 12, *R*_*i*_ is the rank of the *ith* observation in the *X*_*i*_ temporal data and *n* is the total number of observations. Positive values of *Z*_*SR*_ indicate that there is an upward trend, meaning that at particular meteorological stations the wet conditions are increasing with time. Negative *Z*_*SR*_ values indicate a downward trend and that precipitation is decreasing more than normal. When $Z_{SR}>t(n-2,\;1-\frac{\alpha }{2})$, the alternative hypothesis is accepted, meaning there is a significant trend in the temporal data. For case $Z_{SR}<t(n-2,\;1-\frac{\alpha }{2})$, the null hypothesis is accepted and there is no significant trend in the temporal data. The $t(n-2,\;1-\frac{\alpha }{2})$ is the critical value of *t* from the t-student table for a 95% significant level, which is 2.0167 for tow tailed.

## Results and discussion

### Spatial distribution of rainfall and aridity index

**Fig 2:**
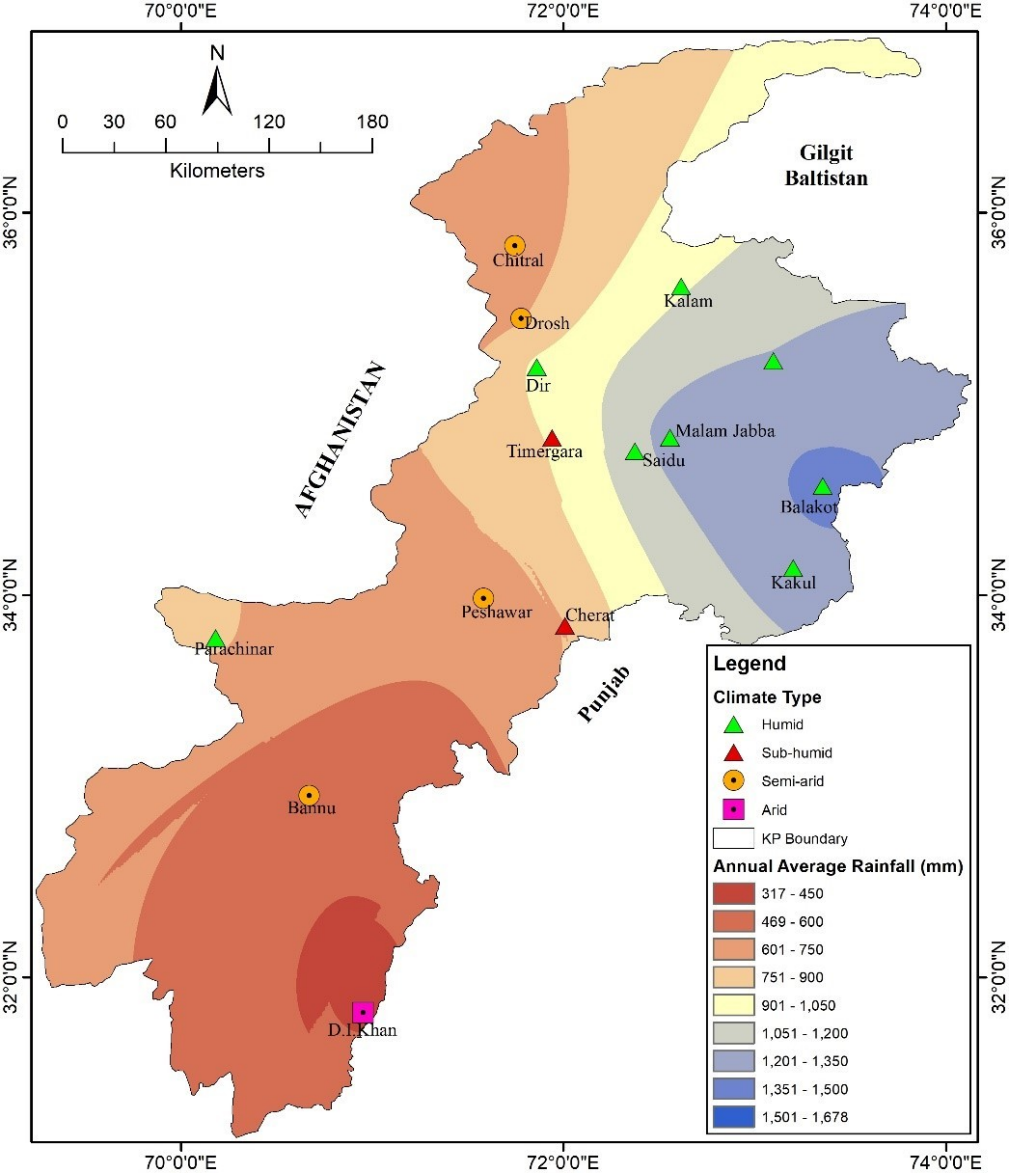
Khyber Pakhtunkhwa, spatial distribution of average annual rainfall and type of climate as per aridity index.

To assess spatial variability in the rainfall and aridity index, the data collected from all 15 meteorological stations were analysed. In this study, aridity index was calculated using P/PET, where PET was calculated using the Thornthwaite method. According to [31] the values in the aridity index are classified as arid climate (0.03 to 0.2), semi-arid (0.21 to 0.5), sub-humid (0.51 to 0.65) and humid areas with an aridity index greater than 0.65. The present analysis revealed that in the study area the aridity index ranges from a minimum 0.18 in the southern region of the province at met-station D.I. Khan to a maximum value of 1.8 at the Malam Jabba met-station located in the northern region of the study area. The meteorological stations Kalam, Malam Jabba, Saidu, Pattan, Kakul, Balakot, Dir, and Parachinar are classified as humid based on the UNEP aridity index (Table 2; Fig 2). The Cherat and Timergara met-stations are sub-humid, while Chitral and Drosh are classified as a semi-arid type of climate according to the aridity index. In the study area, D.I. Khan is the only meteorological station with an arid type of climate.

**Table 2:** Khyber Pakhtunkhwa, geographic and climatic characteristics of met-stations.

*Met-Station*	*Longitude (E)*	*Latitude (N)*	* Elevation (m)*	*Mean annual rainfall (mm)*	*Mean Annual PET (mm)*	*Aridity Index*	*Climate (UNEP 1997)*
*Balakot*	73°21’22"	34°34’15"	1020	1588.94	1407.98	1.128	*Humid*
*Bannu*	70°40’18"	32°57’1"	340	361.92	1690.85	0.214	*Semi-arid*
*Cherat*	72°0’28"	33°50’21"	520	622.39	1124.38	0.553	*Sub-humid*
*Chitral*	71°44’50"	35°49’34"	2130	453.69	1450.93	0.313	*Semi-arid*
*D*.*I*. *Khan*	70°57’10"	31°48’56"	170	316.53	1747.64	0.181	*Arid*
*Dir*	71°51’37"	35°11’33"	1650	1409.48	1388.01	1.015	*Humid*
*Drosh*	71°46’46"	35°26’40"	2230	572.06	1419.45	0.403	*Semi-arid*
*Kakul*	73°12’6"	34° 8’28"	1260	1349.87	1310.75	1.029	*Humid*
*Kalam*	72°36’57"	35°36’51"	4370	1049.09	1144.74	0.916	*Humid*
*Malam Jabba*	72°33’28"	34°49’23"	2650	1683.03	891.07	1.888	*Humid*
*Parachinar*	70°10’54"	33°46’31"	1720	986.87	1273.64	0.775	*Humid*
*Pattan*	73° 6’17"	35°13’54"	2275	1129.88	1522.67	0.742	*Humid*
*Peshawar*	71°33’56"	34° 0’54"	345	457.83	1598.216	0.286	*Semi-arid*
*Saidu*	72°21’38"	34°44’58"	1050	1047.72	1487.65	0.704	*Humid*
*Timergara*	71°50’26"	34°49’33"	1475	802.36	1557.60	0.515	*Sub-humid*

In Khyber Pakhtunkhwa, there is a great variation in altitude, and therefore a large variability in rainfall has been recorded. The highest average annual rainfall was recorded at the Malam Jabba met-station (1683 mm) followed by Balakot (1588.94 mm) and Dir (1409.48 mm). These meteorological stations are located at high altitude and receive rainfall from both the monsoon and western depression. A lower average annual rainfall was recorded at the D.I. Khan (316.52 mm), Bannu (361.9 mm), Chitral (453.69 mm) and Drosh (572 mm) meteorological stations (Table 2).

**Fig 3:**
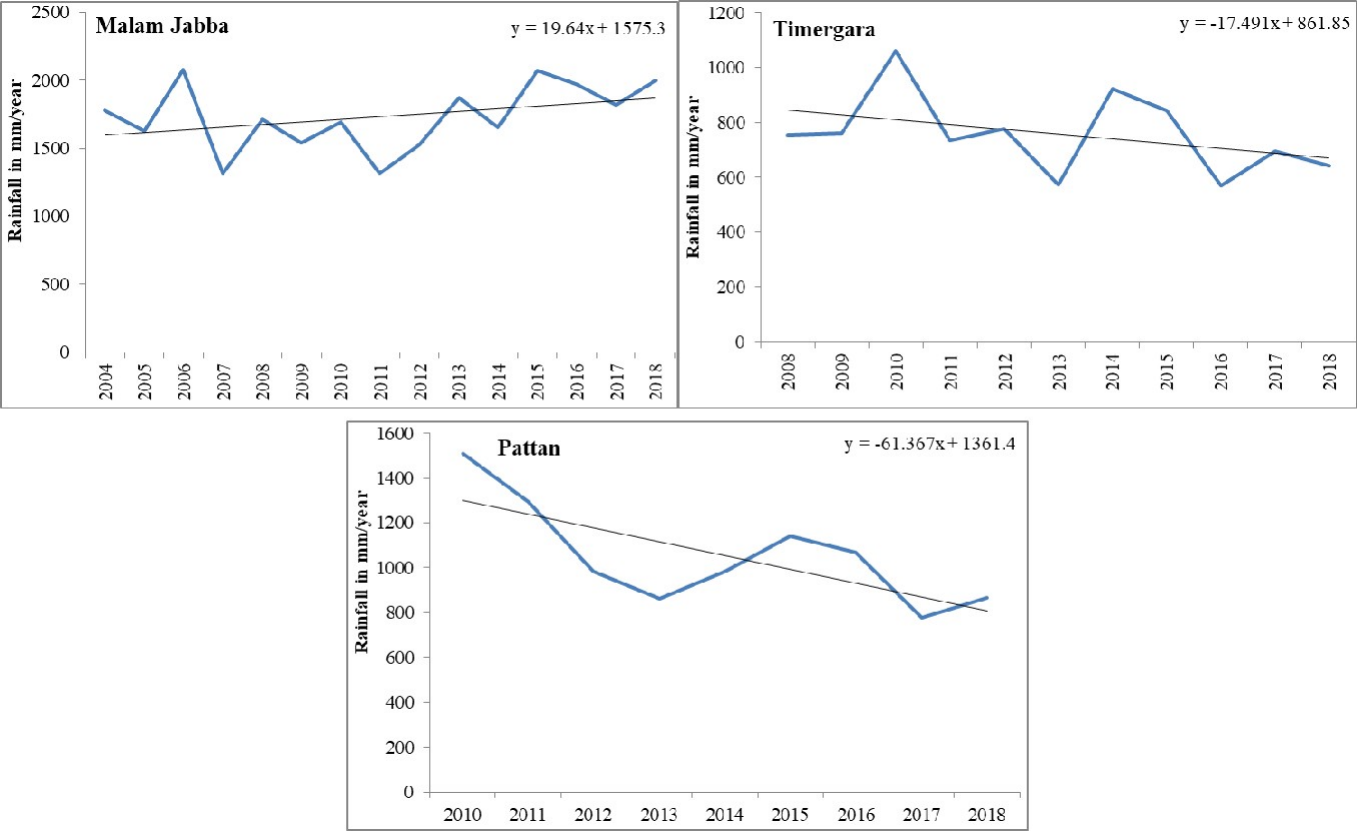
Annual rainfall variation in selected met-station.

The meteorological stations D.I. Khan and Bannu are situated in arid and semi-arid low-lying plains, whereas Drosh and Chitral are located at high altitude on the rain shadow side, and therefore receive less rainfall. Linear regression was calculated for ten of the met-stations to identify changing patterns in rainfall because long term rainfall data was available for these met-stations. Out of the ten met-stations, five (Balakot, Kakul, Dir, Saidu and Drosh) were found to have a negative slope, meaning that in these met-stations the amount of rainfall was decreasing with time (Fig 3). The highest decline in the amount of rainfall was in Balakot with 11.57 mm/annum, followed by Kakul with 4.68 mm/annum and Dir with 3.7 mm/annum. The rate of decrease in the amount of rainfall at these meteorological stations is alarming since these stations are situated at high altitude, and the region has large biodiversity and also hosts major glacial reservoirs. The decline in precipitation due to a changing climatic pattern is a threat to these glacial reserves. Change in the climatic pattern in this region is due to some anthropogenic activities like deforestation, and the extension of built-up areas on the mountainous slopes. The decrease in the amount of precipitation in meteorological stations Saidu was 2.6 mm/annum and Drosh 2.3 mm/annum during 1971–2018 (Fig 3).

Meanwhile, the amount of rainfall in the central and southern part of the province is increasing. The results of linear regression show the highest slope of increase at the Parachinar met-station (18.27 mm/annum), followed by Cherat (2.82 mm/annum), Peshawar (1.85 mm/annum) and D.I. Khan (1.81mm/annum) (Fig 3). There was a consistent increase in the amount of rainfall at the Parachinar met-station after 2004, and an abrupt decrease was recorded there after 2015. This increase in rainfall in the southern part has some correlation with changes in local winds, and can also be partly attributed to the development of a low pressure belt at Bannu and D.I. Khan in summer, which brings rainfall to the Parachinar because of its high altitude.

### Monthly and annual rainfall trend in Khyber Pakhtunkhwa

Rainfall trend in Khyber Pakhtunkhwa has been analyzed on a monthly and annual basis using Spearman’s R on the data of meteorological stations for the period of 1971–2018. The results of monthly data revealed that in January the significant negative trend was only detected at Balakot met-station while in most of the met-stations the rainfall was insignificantly decreasing (Table 3). The February month shows the only significant increasing trend was at Parachinar while the significant decreasing trend was found at Pattan. In March the significant decreasing trend was detected at Balakot and Saidu met-stations and significant increasing trend was at Parachinar. In April only significant increasing trend was detected at Parachinar from SR results. In May the significant increasing trend was again at Parachinar while in June the significant trend was detected at Parachinar, Peshawar and Saidu met-stations. In July the significant decreasing trend was at Balakot and in August this was at Kakul. Both the met-stations (Balakot and Kakul) are located in Himalayan ranges on north-east of the province where most of the rainfall occurs in these months from the monsoon. In September only significant decreasing trend was detected from SR results at Pattan while in October and November the significant increasing trend was found at Parachinar met-station. The alarming situation was found from the results of trend analysis in the month of December as almost all the met-stations recorded negative trend although the significant decreasing trend was only found from the results of SR at Drosh (Table 3).

**Table 3:** Spatial distribution of Spearman’s Rho *Z* value for monthly and annual rainfall.

Met Station	Jan	Feb	Mar	Apr	May	Jun	Jul	Aug	Sep	Oct	Nov	Dec	Annual
Balakot	**-2.12**	-0.98	**-2.13**	-0.3	0.29	0.05	**-2.5**	-1.89	-0.37	0.86	0.18	-1.77	**-3.92**
Bannu	-0.6	-0.14	0.66	0.73	0.4	-0.69	-0.55	0.88	-0.11	0.64	0.98	0.12	0.18
Cherat	0.04	0.34	-0.59	1.99	0.61	1.16	-0.11	-0.51	0.39	1.05	1.25	-0.97	1.7
Chitral	0.21	0.54	-1.18	-0.3	-0.61	1.45	0.41	0.4	0.21	-0.67	1.28	-1.36	0.26
DI Khan	-0.63	0.62	0.35	-0.12	-1.72	1.11	0.77	0.29	0.88	1.59	0.5	-0.99	0.89
Dir	-0.75	0.42	-0.92	-0.87	-0.45	0.93	-1.09	-1.39	-1.44	0.83	1.14	-1.66	-1.76
Drosh	-0.65	0.16	-1.12	-1.18	-1.38	0.22	-0.54	0.14	-0.72	-0.73	1.23	**-2.16**	-0.93
Kakul	-1.7	-1.05	-1.12	0.54	-0.86	0	-0.64	**-3.31**	0.67	0.08	0.37	-1.25	**-2.57**
Kalam	-1.56	-0.59	0.63	0.5	-0.27	-0.4	0.48	-0.55	-0.92	0.05	-0.48	-1.56	**-3.05**
Malam Jabba	-1.25	-0.31	1.83	1.12	1.66	0.1	1.29	0.64	0.43	0.09	-0.4	-1.02	1.36
Parachinar	1.43	**2.99**	**2.23**	**2.05**	**2.84**	1.85	1.82	**3.16**	1.19	**2.86**	**2.33**	-0.73	**3.91**
Pattan	-0.09	**-3.74**	0.73	1.82	-0.27	-0.22	-0.38	0	**-2.07**	-0.31	0.54	0.54	**-2.17**
Peshawar	0.25	0.71	-1.24	1.09	-0.47	**2.29**	1.39	-0.08	0.7	1.32	1.41	-0.65	1.31
Saidu	-0.61	1.39	**-2.48**	0.1	-1.12	**2.3**	-0.05	-0.62	0.06	-0.48	-0.19	-1.41	-0.67
Timergara	-0.11	-0.36	1.27	-0.67	0.75	-0.46	-0.11	-1.86	-0.58	0.27	0.05	0.15	-1.42

Note: The bold values indicate the significant trend in the monthly rainfall. The ±2.01 is the critical value of Spearman’s Rho from the t-student table indicating a significant trend at 95% significance level.

The results of SR trend tests revealed a significant decreasing trend in Kalam, Balakot and Kakul meteorological stations while the Pattan shows a significant decrease in in annual rainfall (Table 3). These all are located on the eastern side of Khyber Pakhtunkhwa. Balakot and Kakul met-stations are in the Himalayas while the Kalam and Pattan are located in Hindu Kush ranges. Here the decrease in the amount of rainfall could create implications of water availability for agricultural and hydro-power generation. Similarly, the meteorological stations Drosh, Dir, Timergara and Saidu also exhibit the same pattern of decreasing trend in the annual rainfall but the results were found insignificant at 95% significance level (Table 3). The only significant increasing trend was observed in Parachinar where the results reveal that annual rainfall is increasing and the results were found significant at 95% significance level in SR trend tests. The meteorological stations Malam Jabba, Peshawar, Cherat and D.I. Khan show an insignificant increasing trend in annual rainfall.

### Spatio-temporal variation of SPEI

**Fig 4:**
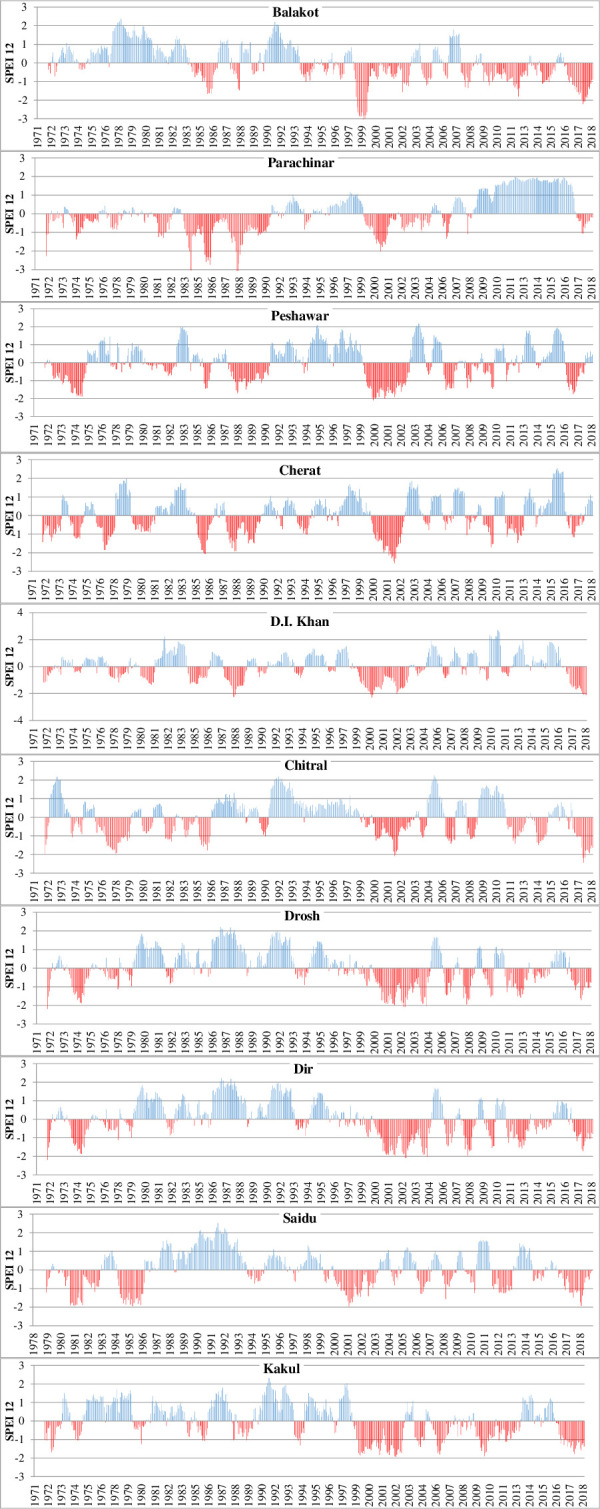
Khyber Pakhtunkhwa, SPEI 12-month. The blue bars represent wet period while the red shows dry.

In this study, the 1-month and 12-month SPEI were calculated for all fifteen metrological stations in the study area. Fig 4 shows the spatial and temporal variation in the 12-month SPEI results. The study results reveal both extreme wetting and drying conditions in the study area during the study period 1971 to 2018.

Between 1971 and 1975, a severe dry condition was observed at the Parachinar, D.I Khan, Peshawar, Cherat, Kakul and Dir meteorological stations in the SPEI 12-month results (Fig 4) and the same was observed in the SPEI 1-month. The drought of 1970–1971 in Khyber Pakhtunkhwa [32] is considered to be the worst drought. This dry spell was followed by an extremely wet period in almost all meteorological stations except Saidu and Parachinar. This wet period brought devastating floods in 1976, 1977 and 1978 in Pakistan that caused the loss of 1666 human lives, and 29,774 villages were affected [33]. The wet period was again followed by drought during 1984–1989. The SPEI values in that period showed severe to extreme drought conditions, mostly at met-stations located in the southern part of Khyber Pakhtunkhwa including Parachinar, Cherat, Peshawar, and D.I. Khan met-stations and Dir met-station in the north. During this period extreme drought was recorded in 1987–1988 [32]. However, the rest of the met-stations recorded moderate to severe drought during 1984–89.

During the study period, extreme drought was observed throughout Khyber Pakhtunkhwa province, from 1998 to 2003. This consecutive drought period resulted in a decline in the groundwater table, causing agricultural crop failure and starvation all over the province. It was the worst drought since the inception of Pakistan, and affected about 3.3 million people in the country. Looking to the SPEI 12-months results, it seems that consecutive dry periods after the 1998–2003 drought period occurred in most of the met-stations like Balakot, Dir and Kakul located in the northern part of the province. This observed increase in drought frequency and intensity during the study period is linked with a decrease in precipitation and rise in temperature over the whole region, which enhanced evapotranspiration and soil moisture drying. Another reason is the decline in the number of cyclones originating from the Bay of Bengal and the Arabian Sea over the last four decades. But despite the reduction in frequency, the intensity and damages caused by these cyclone have risen [34]. The dry spell in 2009 badly affected crop yield in the province of Khyber Pakhtunkhwa [35]. In the years 2017–2018 all the meteorological stations in the province experienced moderate to severe drought.

### Spatial distribution of Spearman’s Rho (SR) trend test

**Fig 5:**
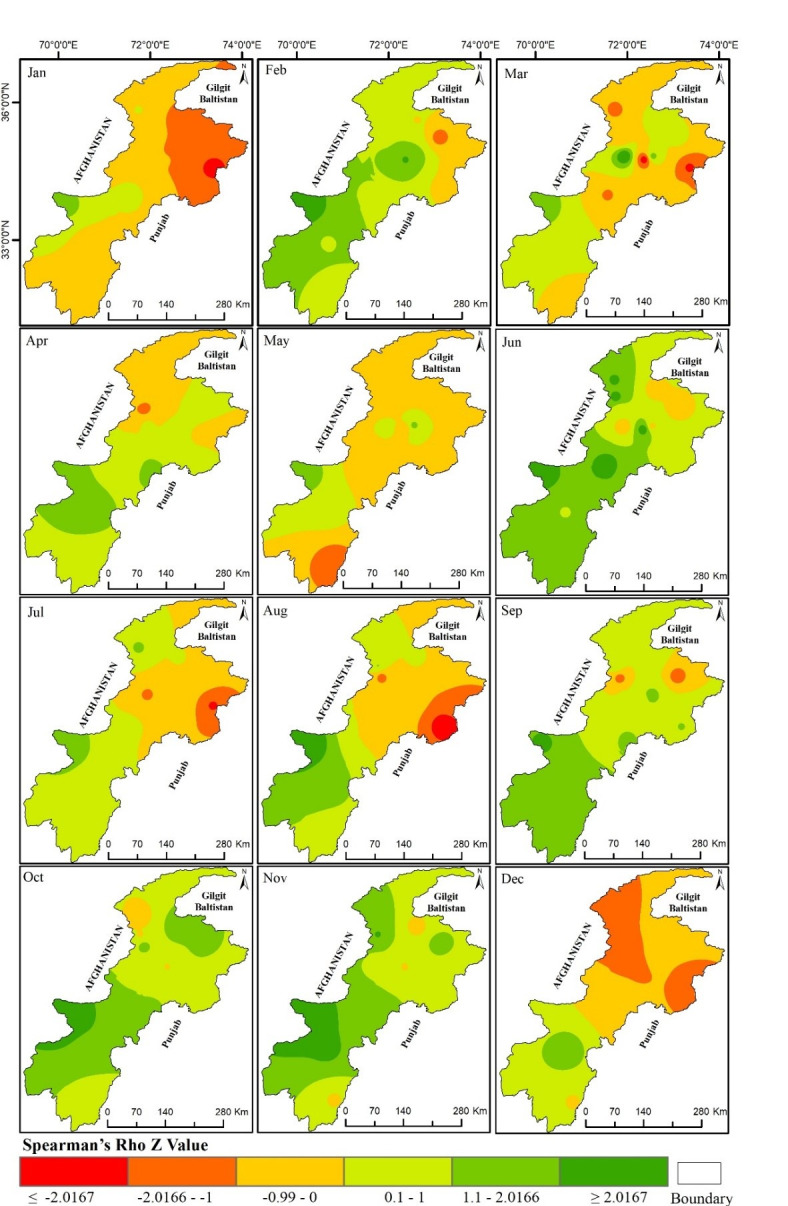
Spatial distribution Spearman’s Rho Z value for SPEI 1-month. (The Z value of ±2.0167 is a significant trend at a 95% confidence level).

The SR trend test was applied to the SPEI 1-month and SPEI 12-month results to determine the drought trend, and were interpolated for spatial analysis. Fig 5 shows the spatial variation in the 1-month SPEI in the province during 1971–2018. The ±2.0167 *Z*_*SR*_ values indicate the existence of a significant trend at the 95% confidence level. The negative *Z*_*SR*_ value means the increase in drought and decrease in precipitation in those particular meteorological stations, while the positive *Z*_*SR*_ value indicates the prevalence of wet conditions.

The SPEI 1-month results showed there was a significant dry trend in January in the north eastern part, and a dry but not significant trend was found in other parts of the province except Parachinar, where the SPEI values were found to be positive (Fig 5). The Spearman’s Rho results for the month of February showed a significant positive trend at Parachinar and a positive but insignificant trend in the maximum at the meteorological stations of the area. This indicates an increase in precipitation in the month of February in the study area. In March, the Balakot and Saidu met-stations exhibited a significant negative trend in the 1-month SPEI results while a positive significant trend was observed at the Timergara met-station. Almost all the other stations except Parachinar and Malam Jabba recorded a dry insignificant trend in the study area.

The Spearman’s Rho results for the months of April and May exhibited no significant trend while the month of June results showed a significant increasing trend in wetting conditions in the meteorological stations Chitral, Drosh, Parachinar, Peshawar and Saidu. The wetting conditions in the month of June reflect an early onset of the monsoon, as stated by S. Ali et al. (2019). The months of July and August witnessed a significant decreasing trend in the north eastern and northern parts of the province. This is also attributed to a decrease in the amount of monsoon precipitation, as concluded by [36]. October and November exhibited an increasing but insignificant trend in the 1-month SPEI results, while in December there was a decrease in the amount of precipitation and an increase in drought conditions.

**Fig 6:**
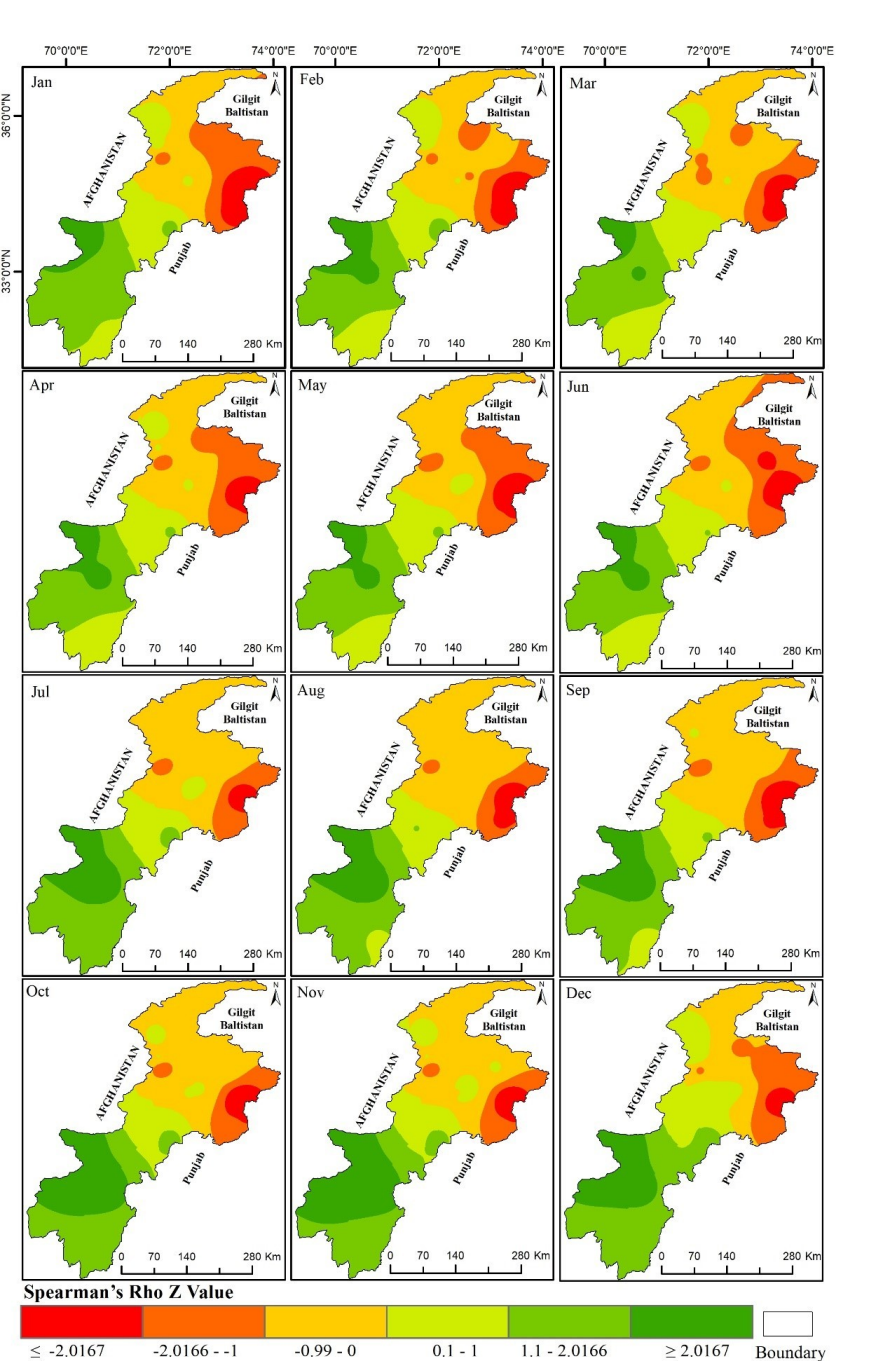
Spatial distribution Spearman’s Rho *Z* value for SPEI 12-month. (The *Z* Value of ±2.0167 is the significant trend at a 95% confidence level).

Fig 6 shows the spatial distribution of the SR trend values in the 12-month SPEI results. These SPEI results are based on the accumulated precipitation for the whole year. The SR trend is shown for each month of the year in Fig 6. The results show a significant negative trend (decrease in precipitation and an increase in drought conditions) in the north-eastern part, and a positive trend (increase in precipitation and increase in wetting conditions) in the south-western part of the province, especially at the Parachinar met-station. Fig 6 indicates there were increasing drought conditions in almost all of the meteorological stations located in the northern part, and increasingly wetter conditions in the southern part of the province.

Looking at the overall results of the study, they indicate that the meteorological stations situated in the north and northeast of the province have recorded a decreasing trend in the amount of precipitation. Since these meteorological stations are situated in humid and sub-humid regions, a small decrease in the amount of precipitation may not have any local impacts, but it is creating an alarming situation for the agricultural sector, and for water resources in the province, as this region is the source of glaciers, and most of the rivers in this region originate from these glaciers. The increasing drought trend in December to March means a decline in the amount of precipitation which will in turn affect the amount of glacier accumulation in these months. This will increase shortages of water available for agriculture as well as for the energy sector.

## Conclusion

This study focused on the spatio-temporal variability of rainfall and drought in Khyber Pakhtunkhwa during 1971–2018. Aridity was measured for each met-station using the UNEP 1997 climate aridity index. SPEI was applied to assess the drought conditions in the study area and Spearman’s Rho trend test was applied to detect trends in the 1-month and 12-month SPEI results. Out of the 15 met-stations, 8 were Humid, 2 were sub-humid, 4 were Semi-arid and one had an arid type of climate. The linear regression results of the rainfall data showed the Parachinar met-station had the steepest increasing trend, with a slope of 20.45 mm/annum while the steepest decreasing trend was at the Balakot met-station with a slope of 10.75 mm/annum.

The results for the 1-month and 12-month SPEI revealed both dry and wet spells in the studied period. The time periods 1971–1974, 1984–1989, 1998–2003 and 2017–2018 were observed to have moderate to extreme drought conditions at almost all meteorological stations in the study area. The results of the Spearman’s Rho trend test indicated a highly significant negative (decreasing precipitation) trend in the north-eastern part of the province, in both the 1-month and 12-month SPEIs. A significant increasing trend was found in data from the Bannu and Parachinar met-stations in the 12-month SPEI results. The overall results of the study revealed that meteorological stations situated in the north and north-eastern part of the study area exhibited a drought trend and decreasing amount of precipitation, while there was an increase in wet conditions in the southern part of the province.

The results of this study will provide a foundation for future studies of drought, precipitation and temperature variability as well as for the study of hydrology in Khyber Pakhtunkhwa. The identification of increasing drought areas can be useful for drought risk management as well as for the sustainable management of water resources. There is an urgent need to devise a system for the mitigation of meteorological disasters in the study area.
